# Phytochemical analysis and broad spectrum antimicrobial activity of ethanolic extract of *Jasminum mesnyi* Hance leaves and its solventpartitioned fractions

**DOI:** 10.6026/97320630014430

**Published:** 2018-08-31

**Authors:** Radha Verma, B. S. Balaji, Aparna Dixit

**Affiliations:** 1 School of Biotechnology, Jawaharlal Nehru University, New Delhi, India

**Keywords:** *Jasminum mesnyi* Hance, Antibacterial activity, Bacterial pathogens

## Abstract

*Jasminum mesnyi* Hance (yellow jasmine, Family Oleaceae) belongs to an important subclass of *Jasminum* family whose biological significance is under explored. The current study pertains to isolation of various solvent extracts fractions and their anti-bacterial effects on the Gram-positive and Gram-negative bacterial pathogens. Ethanolic extract of *J. mesnyi* Hance leaves was subjected to further partitioning using different solvents with increasing polarity to get different solvent fractions. Different extracts were analysed for their phytochemical constitutents and were evaluated for their antibacterial activity against a number of diseases causing bacteria. Diethylether fraction (DEF) showed remarkable inhibition of growth of *Aeromonas hydrophila* and *Vibrio parahaemolyticus* with inhibition zones of 17 and 19mm, respectively at 250 μg. On the other hand, the hexane fraction (HF) was found to be more effective against Gram- positive test bacterial pathogens (*Bacillus anthracis* and *Bacillus subtilis*) with inhibition zones of 19.6 mm and 17.5 mm, respectively. The phytochemical screening of different test fractions revealed the predominant presence of cardiac glycosides, sterols and terpenoids in DEF and HF, while Ethylacetate fraction (EAF) and methanol fractions (MF) were found rich in flavonoids and phenols with moderate amount of other reference metabolites.

## Background

Many human and animal diseases arise due to either bacterial or viral infections. Bacterial diseases top the list of infectious diseases caused by biotic agents. While several such infections may be treated or controlled by antibiotics, their indiscriminate use has resulted in bacterial adaptation to these molecules and antibiotic resistance. Due to emergence of drug resistance, many antibiotics have become less effective against pathogenic bacteria [1, 2]. According to the Centers for Disease Control and prevention, at least 2 million people become infected with antibiotic resistant bacteria every year in the United States only, of which approximately 25,000 people die each year as a direct result of these infections [3].

Thus, emergence of antibiotic resistant strains and adverse effects of the existing antibiotics has become a potential hurdle in the treatment of infectious diseases. This necessitates the search for new antimicrobial molecules/agents that may efficiently overcome the drug resistance problem as well as alleviate the adverse effects of currently employed antibiotics [4]. Natural products derived from various life forms including microorganism, plant, and animal are endowed with excellent properties like chemical diversity, structural complexity, affordability, and lack of substantial toxic effects.

Extracts of various plants and their parts have been used in traditional systems of medicine for treating various illnesses including bacterial infections. Plants derived natural products and their derivatives are known to possess antimicrobial activity against infectious biotic agents namely bacteria, yeast, molds, nematodes, and viruses [5]. These extracts contain various phytochemicals including alkaloids, terpenoids, flavonoids, glycosides, essential oils, phenols etc., which exhibits significant potential in therapeutics and have been used by a number of pharmaceutical industries [6, 7, 8, 9]. Secondary metabolites isolated from natural sources have been shown to elicit broad-spectrum antimicrobial activities and thus have vast scope for therapeutic applications [10, 11]. Owing to their antioxidant and antimicrobial efficacy, low toxicity and cost, plants are the best source to screen for novel antimicrobial agents with minimal adverse effect that are associated with the currently available antibiotics [12].

A number of plant extracts have been shown to exhibit antimicrobial activity in vitro. Systematic fractionation and analysis of various fractions of plant extract is likely to result in a most effective fraction enriched in antimicrobial molecules. This may ultimately lead to identification of novel bioactive compounds possessing antimicrobial activity [13].

Jasminum mesnyi Hance (Family Oleaceae) is commonly known as Japanese jasmine or primrose jasmine. Referred to as pili chameli (yellow jasmine) in hindi, J. mesnyi Hance is an evergreen perennial flowering shrub cultivated in tropical and sub-tropical parts of the world [14] J. mesnyi is originally from China and Vietnam, however, has been naturalized to other parts of the world. In India, it is grown in the Himanchal Pradesh and the Nilgiris region [14]. Many species of Jasminum are well known for their commercial use in perfume industries as well as in folklore medicine [15]. Paste of Jasmine flower has been used for treating sunburns, rashes and for wound treatment [16] Anti-depressant and aphrodisiac activities of Jasminum oil have also been reported [16]. Another species of Jasminum, J. humile (Italian Jasmine), bearing yellow flowers (pili chameli) has been reported to possess a variety of biological activities such as astringent, decongestant, anti-parasitic, stomachic and as a tonic for the heart and bowel [17, 18]. Aqueous root extract J. humile has been found to be effective in the treatment of skin infections [19]. Despite the knowledge of diverse therapeutic properties of its sister species J. humile, few studies have been conducted to assess different bioactivities of J. mesnyi Hance. Phytochemical composition and presence of various glucosides has been reported [20, 21]. Few investigators have reported the presence of a group of phytochemicals, without any relative concentration in different leaf extract [22, 23]. Antioxidant, anti-hyperglycemic, antihelminthic and wound healing activities of J. mesnyi leaf extract have been reported [24, 25, 26]. However, there are no reports on the antimicrobial/antibacterial potential of this plant. Therefore, the primary aim of the present study is to systematically analyze the phytochemical constituents in the fractionated extracts of J. mesnyi Hance leaves, and to investigate their antimicrobial activity against selected bacteria.

## Methodology

Reagents used in the study were of analytical grade and procured from Sisco Research Laboratory, India or Sigma-Aldrich Chemical Co., USA, unless otherwise stated. Reagents for bacterial growth were from Hi Media Laboratories Pvt. Ltd., India.

### Bacterial Strains

Escherichia coli DH5α cells were obtained from GIBCO-BRL, USA. Aeromonas hydrophila (strain EUS112, MTCC#12301) was a kind gift from Prof. Indrani Karunasagar, College of Fisheries, Mangalore. Staphylococcus aureus (MTCC#740), Vibrio parahaemolyticus (MTCC#451) and Bacillus subtilis (MTCC#441) were procured from the Microbial Tissue Culture Collection, Institute of Microbial Technology, Chandigarh. Bacillus anthracis (Sterne34F2 strain) was a kind gift from Prof. Rakesh Bhatnagar, School of Biotechnology, J.N.U., New Delhi [27].

### Collection and processing of plant material

Fresh healthy leaves of J. mesnyi Hance collected from Shimla, Himachal Pradesh, were washed with double distilled water (DDW) and allowed to air dry under shadow (27°C-30°C). The dried leaves were finely powdered and stored in an airtight container at 4°C until further extraction.

The plant material has been authenticated by the Raw Material Herbarium and Museum, National Institute of Science, Communication and Information Resources (NISCAIR) division of the Council of Scientific and Industrial Research, New Delhi (Ref.# NISCAIR/RHMD/Consult/2018/3252-53). A herbarium specimen has been deposited at the RHMD, NISCAIR for future reference.

### Extraction and fractionation

The leaf powder (50 g) was resuspended in 10 volumes (w/v) of 60 % ethanol and extracted overnight in dark with continuous stirring. After separating the solvent from the particulate material, the extraction process was repeated again. The pooled ethanolic fraction thus obtained, was filtered using Whatman filter paper no.1 and evaporated using Speed vac, (SAVANT ISS110 speed vac concentrator, Thermo scientific, USA). The ethanolic extract thus prepared was designated as the parent fraction (PF), of which an aliquot was saved for analysis.

### Solvent-solvent fractionation

The crude ethanolic extract powder was subjected to solventsolvent partitioning by the method of Kupchan and Tsou [28] with minor modifications. The dried ethanol extract powder (20g) was resuspended in 100 mL of DDW by vortexing, and extracted with equal volume of hexane by stirring overnight at room temperature. The two phases were separated using a separating funnel. The hexane layer was collected and the aqueous layer was re-extracted with additional 100 mL of hexane. With the residual aqueous fraction, the similar process was carried out for other solvents in order of increasing polarity index viz., diethyl ether; ethyl acetate; and methanol (90%) to get different solvent fractions. They were then evaporated in vacuo using rotary evaporator to obtain dried powder. The dried powder extracted with the 60% ethanol and with different solvents thereafter, was weighed to calculate the percentage yield with respect to parent ethanolic extract as:

% Extract recovery = [(Wt. of the glass vessel+extract) - (Wt. of the glass vessel)]*100/ amount of ethanol extract.

### Phytochemical screening

Qualitative analysis of various phytochemicals present in the parent ethanolic extract and subsequent solvent fractions were carried out by standard protocol described earlier [29, 30].

### Quantitation of total phenolic contents

Total phenolic content was determined essentially as described earlier by Cho et al. using the Folin-Ciocalteu reagent (FCR), by adjusting the final reaction volumes to 200μL for a microtiter plate assay [31]. The absorbance was measured at 750 nm against the control (water + FCR and Na2CO3 mixture) and a blank containing only the sample and water. Standard curve using gallic acid was used to determine the total phenolic content, expressed as mg of gallic acid equivalent (GAE)/g of the extract.

### Evaluation of antibacterial activity

#### Broth dilution assay

Antibacterial activity of the leaf extract and its fractions was assessed according to the standard methods available for bacterial pathogens [32, 33]. The primary culture was inoculated and incubated at 37°C overnight. Secondary bacterial cultures (1x108 CFU/mL) were grown in Luria bertani (LB) broth for 2 h, and were finally diluted to 1x105 CFU/mL. The initial stock concentration of different test fractions (2 mg/mL) prepared in triple solvent (TS, acetonitirle: dimethyl sulfoxide: dimethyl formamide 1:1:1) were serially diluted in LB broth and added to the bacterial cultures to a final concentrations indicated in the figure legends (in triplicates). The bacterial cells with corresponding volume of TS were included as controls. The cultures were incubated in an incubator at 37°C and absorbance was measured at 600 nm after 3h upto 12h. To calculate percentage inhibition of bacterial growth, the absorbance of the culture treated with test fraction was adjusted using the controls to which corresponding volume of TS was added. The MIC was determined as the lowest concentration of the compound bringing about significant inhibition of growth.

% Growth Inhibition = [OD600 (control well)- OD600 (inoculums with test fraction)]*100/OD600 (control well)

#### Agar disk diffusion assay

Antibacterial activity of different solvent fractions of leaves extract was carried out using disk diffusion method described earlier [33, 34]. The test samples were diluted to different stock concentrations in TS, so as to keep the volume of applied sample constant. Bacterial strains were cultured in Luria bertani broth at 37°C overnight. Bacterial suspension of 1x105 cfu/mL (0.5 McFarland unit) were prepared and 0.1 mL of suspension was spread uniformly on nutrient agar plates. A sterile disc (10 mm in diameter, HiMedia Laboratories Pvt. Ltd., India) impregnated with different concentrations of the test extracts, was placed aseptically and gently pressed to ensure contact onto the surface of the inoculated agar plate. The plates were incubated at room temperature for 1 h to diffuse the test fractions into the medium. Plates were then incubated at 37°C overnight (14-16 h). Post incubation, zones of inhibition was measured as an indicator of activity. Sterile disks impregnated with TS were included as negative control. Each experiment was performed at least three times.

### Statistical analysis

The results of experiments performed were expressed in mean ± SD. All statistical analyses were done using Graphpad prism version 6. Statistical analysis was carried out using two-way analysis of variance (ANOVA). For all statistical analyses, p value ≤ 0.05 was considered significant.

## Results

### Yield of ethanolic extract and solvent-partitioned extracts

Ethanolic extract (PF) prepared from the J. mesnyi Hance leaves gave a total percentage yield of ~35% (w/w of dry J. mesnyi leaf powder). From the parent ethanolic fractions, calculated percentage yield of different fractions viz. hexane (HF), diethyl ether (DEF), ethyl acetate (EAF) and methanol fractions (MF) were determined to be 4.8%, 12.5%, 18%, and 45%, respectively with respect to the amount of processed ethanoic extract.

### Phytochemical screening

Evaluation for the presence of different phytochemical constituents such as alkaloids, sterols, terpenoids, flavanoids, saponins, tannins, cardiac glycosides and phenols in different fractions of J. mesnyi leaf extract is listed in Table 1. As evident, glycosides, sterols and terpenoids were predominantly present in DEF and HF, though some amounts of flavonoids were also present in DEF. The EAF was found rich in flavonoids with moderate presence of other constituents. Methanolic fraction (MF) had same profile as EAF; however, MF was rich in phenols, almost comparable to the PF.

The total phenolic contents in different fractions of J. mesnyi Hance leaf extract ranged from ~45 mg to 62 mg GAE/g of the extracts. Total phenolic contents of the HF, MF, EAF and DEF were determined to be 44.72±2.41 mg, 53.04±2.29 mg, 61.93±1.86 mg and 63.20±5.03 mg GAE/g of the respective extracts.

### Antibacterial activity of fractionated J. mesnyi Hance leaf extracts

**Figure 1:**
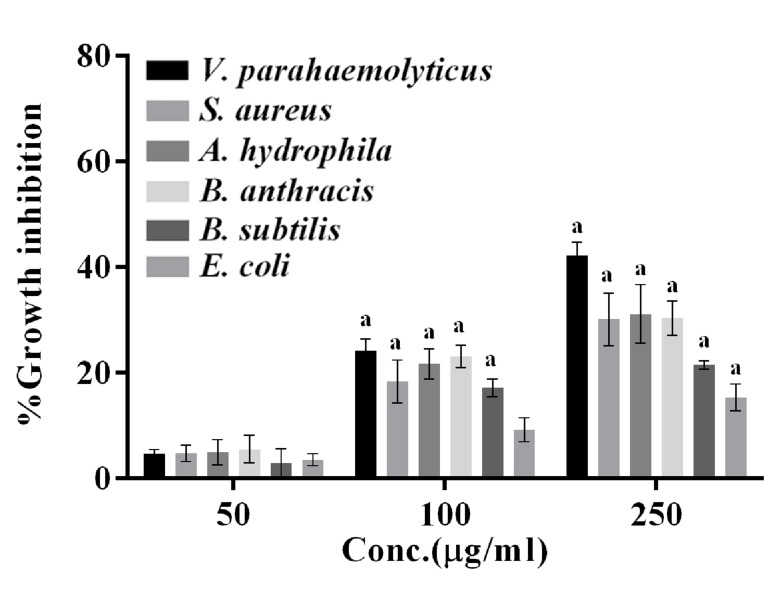
Antibacterial activity of ethanolic extract (parent fraction, PF) of J. mesnyi Hance leaves against different test bacterial pathogens using broth culture assay. The cells were grown in the presence of different concentrations of the PF (50,100, 250μg/ml) or corresponding volume of the vehicle (TS) for 12 h at 37°C. The growth inhibition is expressed as the percentage growth inhibition with respect to control cells treated with TS only. The data represent mean ±SD of three independent experiments performed in triplicates. Significance level of change (p value) is determined with respect to vehicle treated control cells using ordinary two-way ANOVA (Dunette's multiple comparison test). a, p ≤0.0001; b, p ≤0.001; c p ≤0.01; and d, p ≤0.05.

Antibacterial activity of the PF against selected bacterial pathogens was assessed by broth dilution method (Figure 1) at 12 h. An increase in growth inhibition was noted with increase in concentration. The growth inhibition was in the order of V. parahaemolyticus >S. aureus = A. hydrophila = B. anthracis > B. subtilis > E. coli. From the broth dilution assay, the MIC was determined between 50 μg/mL to 100 μg/mL as significant growth inhibition could be observed at 100 μg/mL.

**Figure 2:**
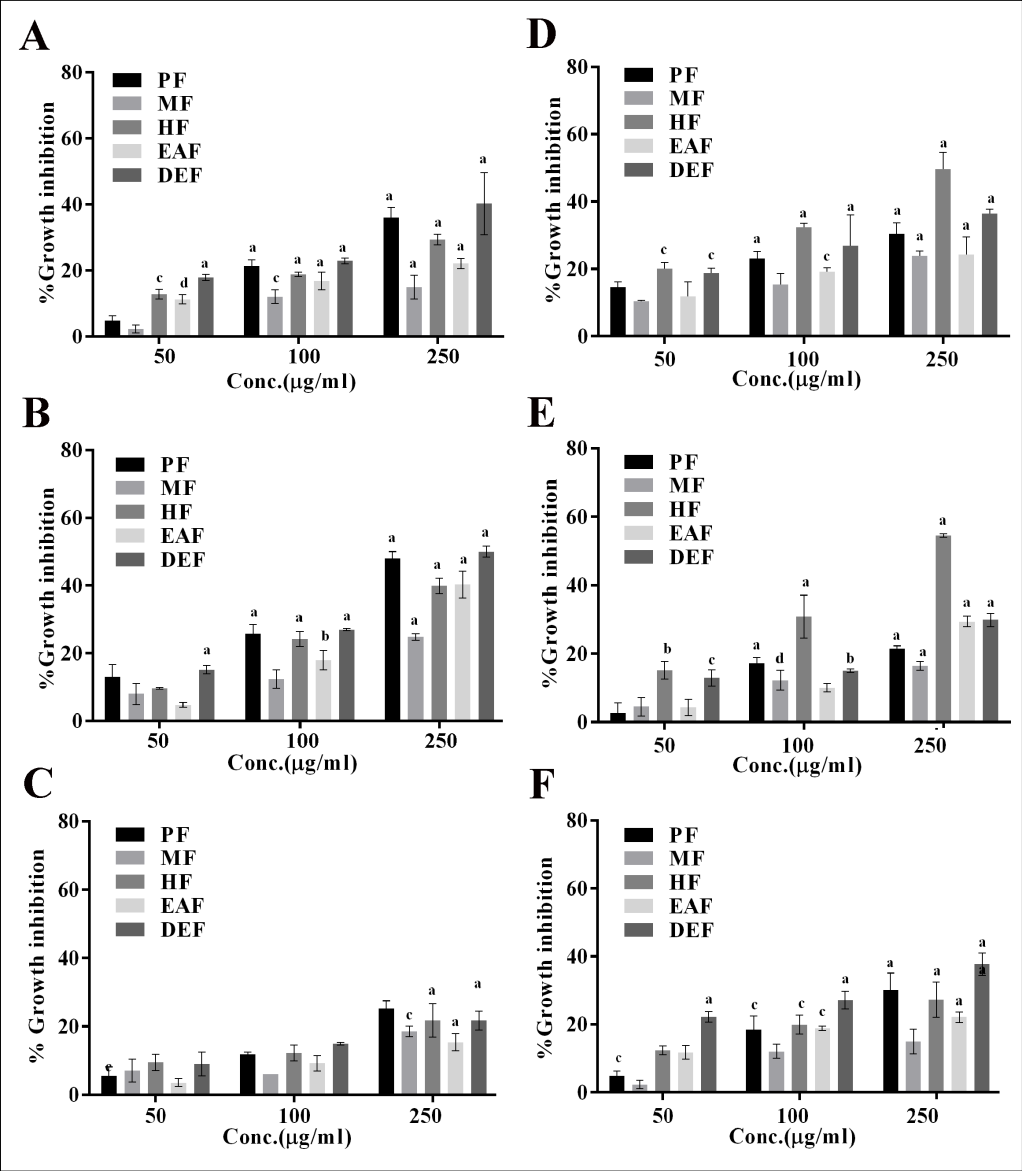
Antibacterial activity of parent fraction (PF) and other solvent-partitioned fractions (HF, DEF, EAF and MF) against different bacteria. Bacterial cells were grown in the presence of varying concentrations of the fractions (50,100, 250 μg/ml) for 12h. The percentage growth inhibition is calculated with respect to control cells grown in the presence of corresponding volume of the vehicle. The data represent mean ± SD of three independent experiments performed in triplicates. Significance level (p value) is calculated with respect to vehicle (TS) treated cells using ordinary two-way ANOVA (Dunette's multiple comparison test). a, p ≤0.0001; c p ≤0.01; d, p ≤0.05. Panels A-C show growth panels of Gram-negative bacteria (A. hydrophila, V. parahaemolyticus and E. coli, respectively, whereas Panels D-F show growth panels of Gram positive bacteria (B. anthracis, B. subtilis, and S. aureus, respectively).

Subsequently, solvent partitioned fractions were assessed for their growth inhibitory ability against the selected bacteria. Though all the fractions showed growth inhibition of A. hydrophila and V. parahaemolyticus (Figure 2A and 2B), the growth inhibition were maximum in the presence of DEF followed by EAF, and minimum in methanol fraction. From these data, the MIC of DEF against V. parahaemolyticus and A. hydrophila were determined to be 50 μg/mL. Growth inhibition in E. coli (Figure 2C) could only be seen at 250 μg/mL. Unlike the above gramnegative bacteria, in which maximum growth inhibition was observed with DEF, maximum growth inhibition of Grampositive bacteria B. anthracis and B. subtilis (Figure 2D and 2E) was brought about using HF with a MIC of 50 μg/mL. While MF, EAF and DEF also inhibited growth of B. anthracis and B. subtilis, a significant increase in growth inhibition was observed with an increase in concentrations only in the presence of HF, suggesting the presence of molecules responsible for growth arrest of these bacteria in HF. In comparison to these two bacteria, relatively lower growth inhibition was observed against S. aureus (Figure 2F).

**Figure 3:**
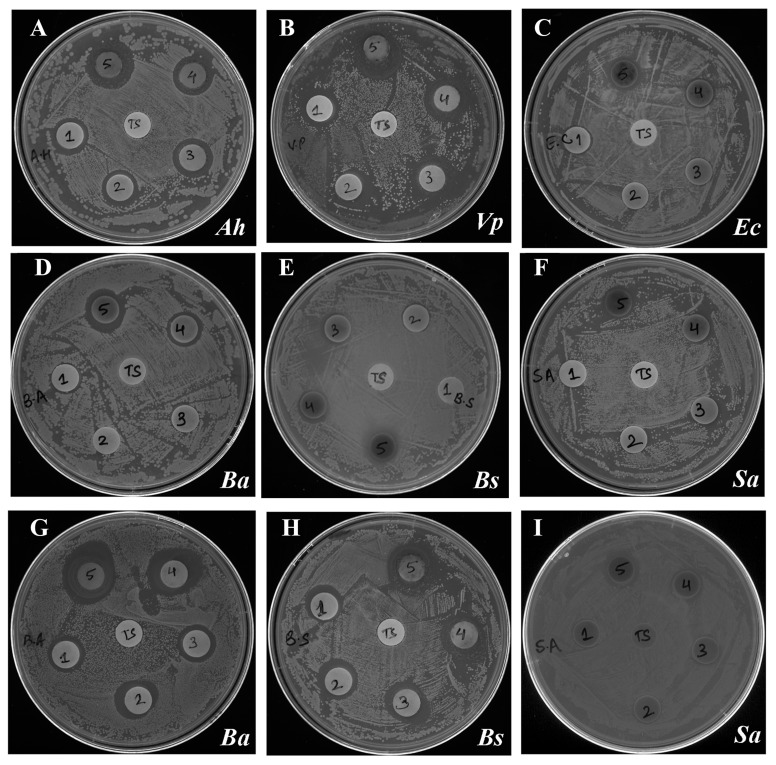
Disc diffusion assay showing growth inhibiton activity represented by zone of inhibition (in mm) of Diethylether fraction (DEF) test fraction against Gram-negative bacteria (A) A. hydrophila (Ah) (B) V. parahaemolyticus (Vp) (C) E. coli (Ec) and Gram-positive bacteria (D) B. anthracis (Ba) (E) B. subtilis, (Ba) (F) S. aureus, inoculated agar plates. Panels "G", "H" and "I" show the effect of hexane fraction (HF) on the growth of B. anthracis, B. subtilis and S. aureus, respectively. Each disk was loaded with 25μg, 50 μg, 100 μg, 250 μg, and 500 μg (1-5, respectively) of the test fraction and incubated overnight at 37°C. In each test place, a control disk loaded with corresponding volume of the vehicle (TS) was included. Disks no. 1-5 represent disks loaded with 25μg, 50 μg, 100 μg, 250 μg, and 500 μg, respectively) of the test fraction. The plates were incubated overnight at 37°C for visualizing the zone of inhibition.

Antibacterial activity of different fractions against all the test bacteria was also determined by discs diffusion assay by measuring the zone of inhibition (mm; including the diameter of disc, Table 2). These results confirmed the findings of broth dilution assay and clearly revealed remarkable potency of DEF against V. parahaemolyticus (Figure 3A) with a zone 19 mm and 21 mm at 250 μg and 500 μg, respectively. This was followed by A. hydrophila with 17 mm and 20 mm at these concentrations of DEF. B. anthracis was found to be more susceptible to HF with mean zone of inhibition 19.6 mm and 22 mm at 250 μg and 500 μg, respectively. In line with the broth dilution assay, DEF at 500 μg showed minimum inhibition zone (12.5 mm) against E. coli whereas HF at 500 μg showed minimum inhibition zone of 15 mm at against S. aureus.

**Figure 4:**
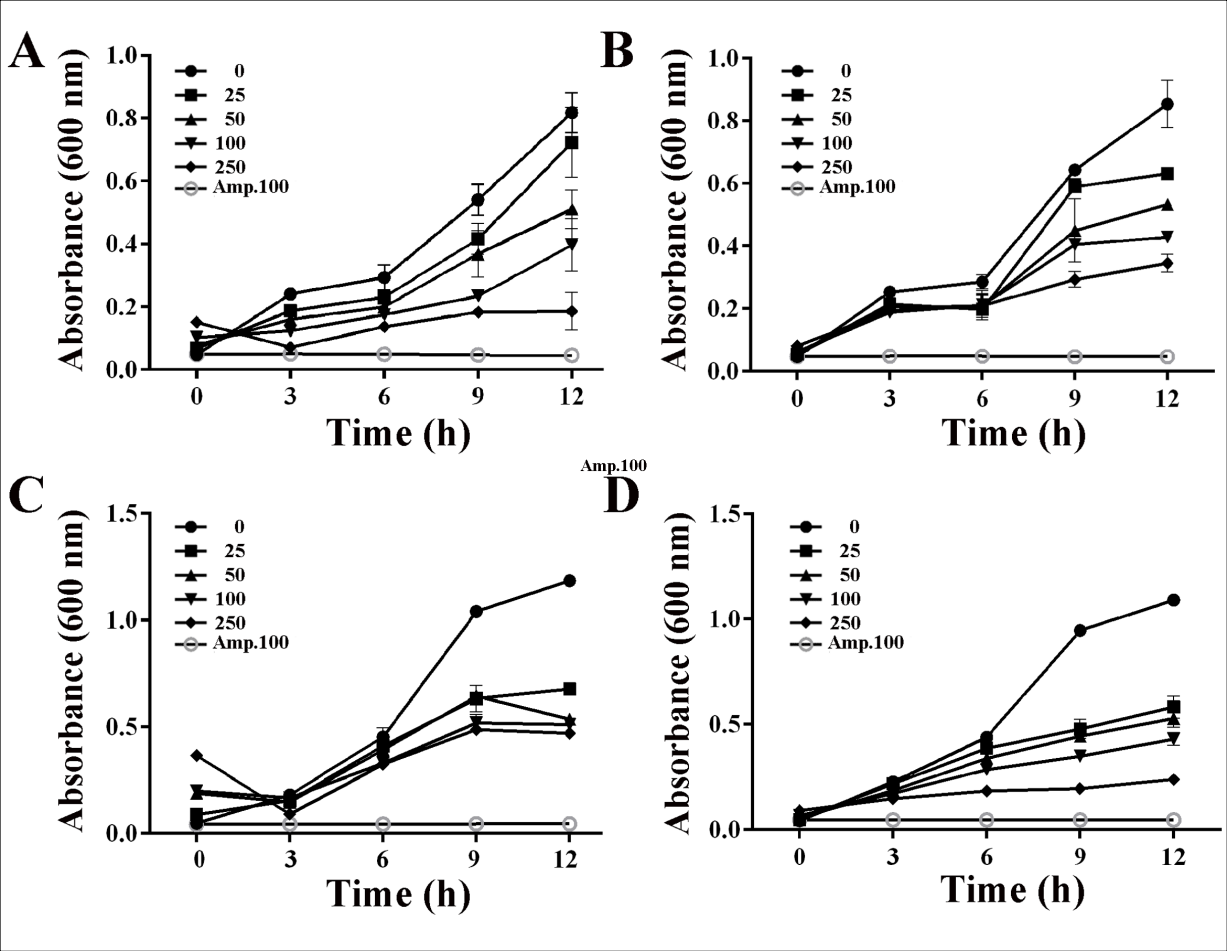
Time and dose kinetics of bacterial growth in the presence of diethyl ether fraction (DEF) and hexane fraction (HF). Growth curves of representative Gram-negative (V. parahaemolyticus, Panels A and C) and representative Gram-positive bacteria (B. anthracis, Panels B and D) grown in the presence of different concentrations of the test fractions (0, 25, 50, 100, 250 μg/mL). The cultures were grown at 37°C and the growth was monitored by measuring absorbance every 3 h upto 12 h. Panels A and B show the cultures grown in the presence of DEF whereas panels C and D show the cultures grown in the presence of HF. Cultures grown in the presence of ampicillin (Amp, 100 μg/mL) were included as a positive control in each case. The data represent mean ±SD of three independent experiments performed in triplicates.

Since DEF and HF showed maximum inhibition of growth of V. parahaemolyticus and B. anthracis, respectively, growth kinetics of these two bacteria in the presence of different concentrations of these two fractions was followed to determine the concentrations and time at which visible growth inhibition could be observed. From these data, it could be concluded that apparent inhibition of V. parahaemolyticus (Figure 4A) growth could be observed as early as 6 h in the presence of DEF. An increase in growth inhibition was seen with an increase in concentration. At 9 h of growth, the IC50 was determined to be 50 μg/mL of the extract. DEF was found to be less effective against B. anthracis (Figure 4B) with apparent significant growth inhibition only at 9 h in the presence of concentrations ≥100 μg/mL. No increase in growth inhibition was seen with an increase in the concentration, suggesting that this fraction contained the molecules in limiting amount. The HF brought some growth inhibition of V. parahaemolyticus (Figure 4C). However, no significant decrease in absorbance was observed with an increase in concentration from 25 μg/mL to 250 μg/mL even at 12 h. On the other hand, the HF significantly inhibited the growth of B. anthracis in a dose dependent manner with an IC50 of 25 μg/mL at 9 h of growth (Figure 4D). At higher concentrations, the growth inhibition could also be seen at 6 h.

## Discussion

Traditional system of medicine has since long relied on the use of extracts of different parts of medicinal plants. These plants confer various activities due to presence of certain phytochemicals. Extracts of different Jasminum species and jasmonates have been reported to have a variety of therapeutic properties [35, 36, 37, 38]. However, unlike other Jasminum species plants, J. mesnyi Hance has not been evaluated extensively except for its few biological activities [24, 25]. Though, antibacterial activity of J. humile (another yellow jasmine) has been reported [19], no reports have been put forth on the antibacterial activity of J. mesnyi. Therefore, it was of interest to systematically fractionate and evaluate its phyto-constituents and assess its antibacterial activity against the selected bacteria.

Secondary metabolites present in plants are sythnesized in response to different stress conditions [39]. Few of these secondary metabolites, including flavonoids and other polyphenols are common naturally occurring antioxidants that have been reported to confer a variety of biological activities [40]. Plants rich in polyphenols have been shown to exhibit both antioxidant and antimicrobial properties [41]. Though few Secoiridoid glucosides have been isolated and characterized from J. mesnyi Hance, systematic analysis of its phytoconstitutents has not been carried out. So far, only few reports have been put forth on composition and volatile constituents of essential oil extracted from the J. mesnyi H leaves grown in Nepal [23]. It is known that phytochemical composition of a given plant varies depending on the immediate environment (soil and water), it was necessary to carry out systematic analysis of extracts of J. mesnyi Hance leaf from India. The qualitative screening of different fractions of leaf extract for phytoconstituents revealed the predominant presence of secondary metabolites such as alkaloids, sterols, terpenoid, glycosides in its DEF and HF while EAF and MF were found to be rich in flavonoids and tannins. The presence of these phytochemicals (flavonoids, glycosides and phenols) in leaf extract of J. mesnyi Hance is in line with its reported antioxidant and antihyperglycemic activities [24]. Ahmed and Beg [42] screened a large number of Indian medicinal plants for their antimicrobial activities and found that the active extracts were rich in phytocompounds including phenols, tannins and flavonoids as major active constituents. Thus, the presence of considerable high concentrations of phenolic compounds (~45-65 mg GAE/g of the extract) together with, glycosides as well as terpenoids in fractionated J. mesnyi Hance leaf extracts indicate their potential as antimicrobial agent. Borar et al. reported only traces of flavonoids in ethyl acetate fraction obtained from methanolic leaf extract of J. mesnyi Hance [24]. The difference in the flavonoid levels in EAF in the present study and that reported by Borar et al. [24] could be attributed to the extraction procedure employed in the two studies.

The parent ethanolic fraction inhibited the growth of all the test bacteria, thus showing its potential as an effective antibacterial agent. Different solvent extracts also showed growth inhibitory properties against a broad range of bacteria including both Gram- negative and Gram-positive, though to a varying degree. Among various test fractions, DEF showed significant growth inhibition in all test organisms, with maximum and minimum inhibition of V. parahaemolyticus and E. coli growth, respectively. The HF was found to be more effective against Gram-positive bacteria in comparison to Gram-negative bacteria. Our results are in agreement with that reported by Campos et al. that hexane extract of Piper solmsianum C. DC. var. solmsianum (Piperaceae) significantly more effective against Gram-positive bacteria (MIC range - 10μg/mL to 100μg/mL) in comparison to gram-negative bacteria (MIC > 1000μg/mL) [43]. The varying degree of inhibition of bacterial growth by different extracts could be due to the presence of different phytocompounds in these extracts. Growth inhibitory effect of the fractionated J. mesnyi Hance leaf extracts on Gram-negative test bacterium V. parahaemolyticus and A. hydrophila is of significance as gram negative bacteria are more resistant towards antibiotics compared to Gram-positive bacteria [44]. Also, the presence of flavonoids and phenols in different fractions, that are known to possess antioxidant activity, in the test fractions advocates its promising use in developing cost effective therapeutic bioactive molecules/preparations with minimum side effects to cure various ailments.

## Conclusion

The present study demonstrates the broad-spectrum antibacterial activity of ethanolic extracts and its solvent partitioned fractions of J. mesnyi Hance leaves. The active extracts were found to be predominantly rich in flavonoids and phenols, correlating well with their growth inhibitory potential against the test bacteria. These extracts can be further tested against other drug resistant bacteria. Further purification of effective antibacterial solvent fractions could lead to identification of an antimicrobial molecule that could be used to treat infections caused by the test organisms.

## Figures and Tables

**Table 1:** Preliminary phytochemical screening of ethanolic extract (PF) of *J. mesnyi* Hance leaves and its different solvent fractions

Extract	Alkaloid	Flavonoids	Glycosides	Sterols	Terpenoids	Saponins	Phenols
PF	+	++	++	+	+	+	++
HF	+	+	++	++	+	-	+
DEF	+	+	++	++	++	-	++
EAF	+	++	+	+	+	-	++
MF	+	++	+	+	+	-	+
PF, parent ethanolic extract; HF, hexane fraction, DEF, diethyl ether fraction, EAF, ethyl acetate fraction and MF, methanol fraction. +, moderate presence; ++, predominant presence; -, absent.							

**Table 2:** Growth inhibitory zone of different extracts of J. mesnyi Hance leaves against different bacteria using disc diffusion method.

Bacterial Strains	Conc. (μg)	Zone of inhibition (mm)	
Gram positive		DEF	HF
*B. Anthracis*	25	10	14
	50	11	15
	100	13	15
	250	14	19.6
	500	15.5	22
*B. subtilis*	25	10	14
	50	10.5	14.5
	100	11.5	15.5
	250	13	17.5
	500	15.5	21.5
*S. aureus*	25	10	10.5
	50	10	11
	100	11	12
	250	12.5	13.5
	500	14.3	15
Gram negative			
*V. parahaemolyticus*	25	16.5	n.d.
	50	16.7	n.d.
	100	19	n.d.
	250	19	n.d.
	500	21	n.d.
*A. hydrophila*	25	16	n.d.
	50	16	n.d.
	100	16.7	n.d.
	250	17	n.d.
	500	20	n.d.
*E. coli*	25	10.5	n.d.
	50	10.5	n.d.
	100	10.7	n.d.
	250	11	n.d.
	500	12.5	n.d.
DEF - Diethyl ether fraction; HF - Hexane fraction; n.d - not determined.
